# Evaluation of the Thermal Insulation Properties of Composites with ZrO_2_/Al Coatings Intended for the Construction of Protective Gloves

**DOI:** 10.3390/ma18020242

**Published:** 2025-01-08

**Authors:** Pamela Miśkiewicz, Adam K. Puszkarz, Marcin Makówka

**Affiliations:** 1 Institute of Architecture of Textiles, Faculty of Material Technologies and Textile Design, Lodz University of Technology, 116 Żeromskiego Street, 90-924 Lodz, Poland; 2 Textile Institute, Faculty of Material Technologies and Textile Design, Lodz University of Technology, 116 Żeromskiego Street, 90-924 Lodz, Poland; 3 Institute of Materials Science and Engineering, Faculty of Mechanical Engineering, Lodz University of Technology, 1/15 Stefanowskiego Street, 90-537 Lodz, Poland; marcin.makowka@p.lodz.pl

**Keywords:** thermal insulation, contact heat, radiant heat, flame heat, protective gloves, basalt fibers, ZrO_2_/Al coating, PVD magnetron sputtering, micro-CT

## Abstract

The article presents research on the evaluation of the use of two four-layer textile composites with ZrO_2_/Al coatings of different thicknesses (deposited by magnetron sputtering PVD) with potential use in thermally insulating protective gloves designed for steelworkers, welders, or miners. The structure of the composites was analyzed using high-resolution X-ray micro-CT. The assessment of the safety of the glove user was conducted using methods in which the composites were exposed to contact heat, radiant heat, and flame heat. The results showed that both four-layer textile composites equipped with ZrO_2_/Al coatings provide effective protection against contact heat, radiant heat, and flame heat and can be successfully used in the construction of metallurgical protective gloves. Both composites achieved the first performance level (for contact heat method, for contact temperature 100 °C), the fourth performance level (for radiant heat), and the third performance level (for flame heat).

## 1. Introduction

A hot work environment occurs when the air temperature is 25–60 °C and relative humidity is 10–80%. The human body in a hot environment releases heat to the environment, but the amount of heat transferred may be insufficient to maintain a constant temperature inside the body. Studies conducted so far have confirmed that long-term work in hot temperature conditions can cause fatigue and heat stress and lead to occupational diseases. In addition, continuous stay in a hot environment leads to dilation of peripheral blood vessels, increased blood flow, increased skin temperature, increased internal body temperature, and increased sweating, which can cause fainting, dehydration, and heat stroke [1,2,3].

Due to the very harmful effect of long-term work in a hot environment on the proper functioning of the employee’s body, it is necessary to thoroughly identify hot workstations and take action to reduce the thermal load on employees. The number of dangerous accidents increases with the increase in the temperature of the work environment and its intensity. Many thousands of people in the world perform their work in hot temperature conditions, e.g., in the metallurgical, glass, and mining industries, or in the summer during heat in the open air [1,4].

In a hot work environment, the employee is exposed to hazardous factors such as direct contact with hot objects, splashes of molten glass and metal, strong thermal radiation, splashes of hot glass, metal, and slag, and the possibility of contact with flame [1,2,4].

Hand injuries are the most common type of injury experienced during work. It is impossible to completely avoid hand injuries during work, because in more than 90% of cases these injuries occur as a result of accidental human error. To some extent, the occurrence of hand injuries can be reduced by using the recommended protective gloves at a specific work station [5].

Basalt is a solid volcanic rock with a dark gray or black color [5,6]. Basalt fibers are referred to as man-made mineral fibers. In order to produce continuous basalt fibers with a diameter of 9 to 24 µm, stones with a constant composition should be used [7,8]. Basalt fibers and products made from them show good thermal resistance from −260 to 800 °C, low moisture absorption, and low thermal conductivity. In addition, basalt fibers are characterized by good resistance to chemicals. Due to their non-flammable properties, basalt fibers and products made from them are resistant to flames for a very long time. In addition, they are resistant to microorganisms, UV radiation, and corrosion. A significant advantage of basalt fibers is their lower price compared to the price of aramid, carbon, and glass fibers [8,9,10,11].

Currently, basalt fibers are produced in the form of filaments that do not cause carcinogenic effects and are not harmful to humans, because only filaments with thicknesses greater than 5 µm are subjected to the spinning process [6,7,12]. Currently, basalt fibers are used to produce specialized products. Basalt fabrics are used to produce composites and are also used to reinforce concrete. In addition, fabrics made of basalt fiber yarn can be used as acoustic insulation, fireproof curtains, sound-absorbing barriers, and thermal insulation [11,12,13]. They are also used as road surface reinforcements, while geotextiles and nets made from them are used in construction for concrete structures. Due to the property’s characteristic of basalt fabrics, they are suitable for use in products protecting against high temperatures and heat, i.e., protective coats, protective clothing, helmets, and protective gloves [12,13,14,15].

One of the increasingly used materials in protection against the harmful effects of hot environments is zirconium dioxide (ZrO_2_) and aluminum (Al). ZrO_2_ belongs to the group of ceramic materials and is most often used in the form of thermal insulation coatings. ZrO_2_ is characterized primarily by low thermal conductivity *λ* (from 1.7 to 2.7 W·m^−1^·K^−1^) [16].

ZrO_2_ is called “ceramic steel” because it combines high wear and corrosion resistance with one of the highest fracture toughness of all ceramic materials [17]. In addition, it is characterized by very high resistance to crack propagation and high fracture toughness (from 6.5 to 8 MPa·m^0.5^) [18]. On the other hand, aluminum, as a material characterized by a low emissivity coefficient ε (from 0.02 to 0.09), is commonly used in the form of coatings as an external barrier reflecting incident thermal radiation. The relatively low emissivity coefficient also makes Al a suitable product for limiting the heat radiated from the human body. For example, it is common to use aluminum coatings in rescue and survival equipment for outdoor operations [19].

The processes of physical deposition of coatings from the gas phase are inextricably linked to the development of vacuum technology, using in their basic versions two basic methods related to the change in the state of the coating material, namely evaporation, or sublimation, and sputtering occurring under the influence of other physical forces other than thermal ones. The functional properties of the deposited coatings depend on individual physical properties and proper adhesion to the substrate. It is difficult to obtain a uniform thickness of coatings that are vacuum sputtered, especially for objects with complex shapes. One of the most important properties of coatings obtained using PVD processes is their adhesion to the substrate. If the adhesion is improper, the functionality of the coating may be lost [20,21,22].

Currently, the most important aspect related to clothing is to provide the most favorable conditions for the user. These conditions are related to their life and work because a person displays intellectual and manual abilities in optimal conditions of thermal comfort. The most important goal of using modern and diverse materials in protective clothing is to maintain the thermal balance of the wearer [1,4,13].

Hrynyk and Frydrych were involved in research on a similar subject, which consisted of developing gloves made of aluminized basalt fabrics that would protect the user’s hands in a hot work environment. Six variants of protective gloves were produced. Aluminized basalt fabrics were used in the back of the glove, but in one of the variants, it was used in the palm of the glove. The aluminized foil used for the tests was a thin, several-micrometer layer of aluminum applied to both sides of a polymer foil. The basis was a foil made of polyester, on the surface of which aluminum was sputtered; the final thickness of the aluminized foil was 12 µm. The method of connecting aluminized foils with fabrics was based on connecting the layers using adhesives under specific temperature conditions. Fulfillment of the requirement for contact heat for a contact temperature of 100 °C was achieved for all variants of the gloves. The use of aluminized basalt fabrics on the back of the glove allows for achieving the fourth level of protection against radiant heat and increases the effectiveness of resistance to small metal splinters [4,7].

The article presents research on two textile composites with original compositions with potential use in thermal protective gloves, on the outer surface of which a two-layer coating consisting of zirconium dioxide (ZrO_2_) and aluminum (Al) was applied by PVD magnetron sputtering that was specially optimized for this purpose, and is a continuation of the research of the authors concerning the safety and ergonomics of protective clothing [23]. The main objective of the research was to find out how this ceramic–metal coating applied in two different thicknesses to the outer surface of composites would affect their thermal insulation properties in terms of the effectiveness of gloves protection against contact heat, radiant heat, and flame heat. In order to investigate the influence of the structure of both composites on their thermal insulation properties, high-resolution X-ray tomography (micro-CT) was used. Micro-CT was used to identify and visualize the spatial geometry of all layers constituting both composites. The porosity of all components and the total porosity of the composites were calculated. Particular attention was paid to the characteristics of ZrO_2_/Al coatings, for which the thickness, porosity, continuity, and uniformity on the substrate on which they were applied were calculated.

## 2. Materials and Methods

### 2.1. Materials

**Figure 1:**
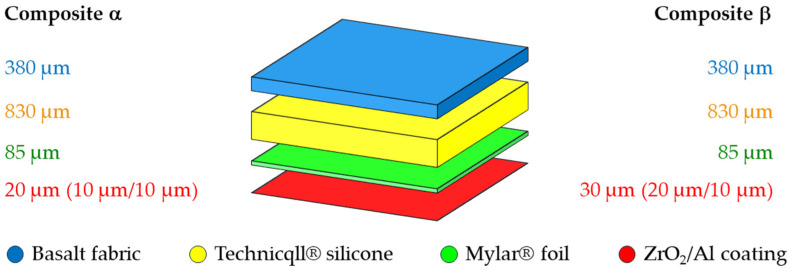
Thickness and mutual arrangement of the layers in both composites.

The subjects of the study were two four-layer composites, α and β, with potential use in thermal protective gloves. The construction scheme of both composites, considering the mutual position and thickness of the layers, was shown in Figure 1 and in Table 1.

Both composites were composed of basalt fabric, HT sealant silicone (Technicqll^®^, Trzebinia, Poland), and Mylar^®^ polyester foil (Du Pont, Wilmington, DE, USA), on which a ceramic–metal coating (ZrO_2_/Al) of various thicknesses was applied, depending on the selected composite.

The selection of materials for the construction of tested composites resulted from their potential use in thermal protective gloves. Therefore, all components are characterized by high resistance to hot temperatures, at which they retain their functional properties that affect the safety and ergonomics of their user in a typical work environment. Fabric made of basalt fibers, which are characterized by high resistance to extreme temperatures, can be successfully used in the temperature range from −260 °C to +800 °C. The thermal conductivity of basalt fibers is in the range from 0.031 W m^−2^ K^−1^ to 0.038 W m^−2^ K^−1^. In addition, these fibers are also characterized by high mechanical strength. The silicone layer acts as a binder that connects the basalt fabric with the Mylar^®^ foil. In both composites, the silicone layer constitutes about 63% of the composite thickness and is also characterized by a low thermal conductivity coefficient (0.14 W m^−1^ K^−1^), and therefore plays a significant role as a thermal barrier. Mylar^®^ foil, due to its appropriate geometry and flexibility, functioned as a smooth and rigid substrate for the ZrO_2_/Al coating applied to its external side. The inner layer of this ceramic–metal coating (ZrO_2_), having different thicknesses depending on the given composite (10 µm in composite α, 20 µm in composite β), due to its low thermal conductivity, acted as an additional heat-insulating layer and was the direct substrate of the outermost layer of the composite made of Al. The Al layer of ceramic–metal coating had the same thickness in both composites (10 µm) and functioned as a reflector for part of the infrared radiation incident on the external surface of the composites, thus constituting a barrier protecting against heat transfer to their internal layers.

### 2.2. Methods

#### 2.2.1. ZrO_2_/Al Coating Method

**Figure 2:**
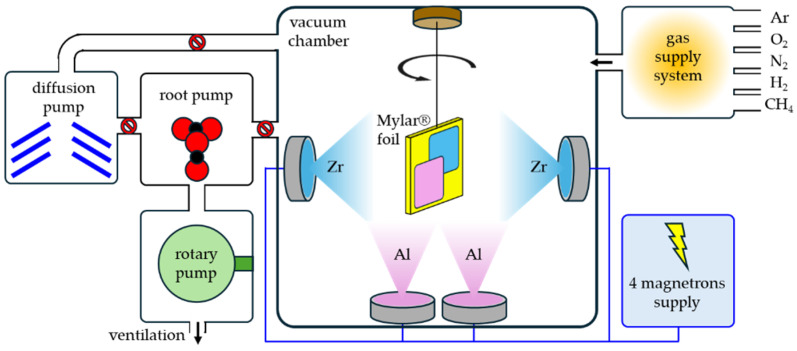
Scheme of ZrO_2_/Al coating method by PVD magnetron sputtering.

ZrO_2_/Al coatings were deposited in a vacuum chamber of the B-90 deposition apparatus (from Hoch-Vacuum, Dresden, Germany) by magnetron sputtering (the scheme is presented in Figure 2).

Four independent planar magnetrons, WK100 (from Dora POWER SYSTEMS, Wrocław, Poland), fitted with circular targets (diameter: 100, thickness: 10 mm), were used. Two targets were made of pure Al (4 N) for deposition of Al coating and two targets from pure zirconium (3.5 N) for deposition of ZrO_2_ coatings. Magnetrons were fixed in the vacuum chamber every 90 degrees in the horizontal plane, and their axes were intersecting in one point. The working gas was argon (5 N), which was used for sputtering the targets and obtaining the material vapors that were the source material of the coating, while pure oxygen (5 N) was additionally supplied when the reactive process of zirconium oxide deposition was carried out.

All substrates before mounting in the vacuum chamber were cleaned in three steps: at first in mild water with detergent, next rinsed in deionized water, and finally in isopropyl alcohol. After mounting the specimens in the specimens’ holder, the vacuum chamber was closed and pumped down to a pressure of 2 × 10^−3^ Pa using a pump system comprising a rotary, root, and diffusion pump. After achieving appropriate residual pressure, the deposition process was conducted according to the following scheme: sample holder rotation at 8 rpm was switched on, and argon was introduced into the vacuum chamber. The pressure of pure argon in the range of 0.46–0.47 Pa was stabilized at a flow of 25 sccm, and then the appropriate magnetron power sources were switched on and a pure Al coating or pure Zr interlayer was deposited. For the reactive processes of the deposition of the ZrO_2_ coating, pure oxygen was introduced into the vacuum chamber with a flow of 25 sccm. Total pressure of argon and oxygen during the reactive process was in the range of 0.47–0.49 Pa. For the deposition of Al, an effective power of value of 1 kW was set on every magnetron with an Al target, while 1.5 kW was set on every magnetron with a Zr target during deposition of the pure Zr interlayer and ZrO_2_ coating. Technological processes were developed empirically, taking into account both the parameters of the magnetron discharge, power, circulating power, discharge current, and BIAS, but also the flow of working gasses like Ar or O_2_ and the time of deposition. The deposited coatings are the result of technological processes with optimized parameters to achieve both the appropriate chemical composition of the individual coatings, their good mutual adhesion, and assumed thickness. Knowing the nature of magnetron sputtering, its disadvantages and limitations, the geometry of the chamber and the arrangement of the magnetrons, and the distance of the holder designed specifically for fabric samples in relation to the magnetron sources were taken into account, and the mentioned technological parameters were selected on the basis of trial processes. In addition, in the case of reactive magnetron sputtering processes, the target poisoning effect is important. The key parameter was to achieve stable target sputtering in mode without a poisoning effect and good Zr/O stoichiometry. This was achieved by monitoring and controlling the circulating power of the discharge on magnetrons with Zr targets and controlling the flow of O_2_. Learning about these phenomena and the influence of the aforementioned parameters made it possible to achieve reproducibility of the technological processes of ZrO_2_/Al coating deposition.

#### 2.2.2. Assessment of Structural Properties of Composites

**Figure 3:**
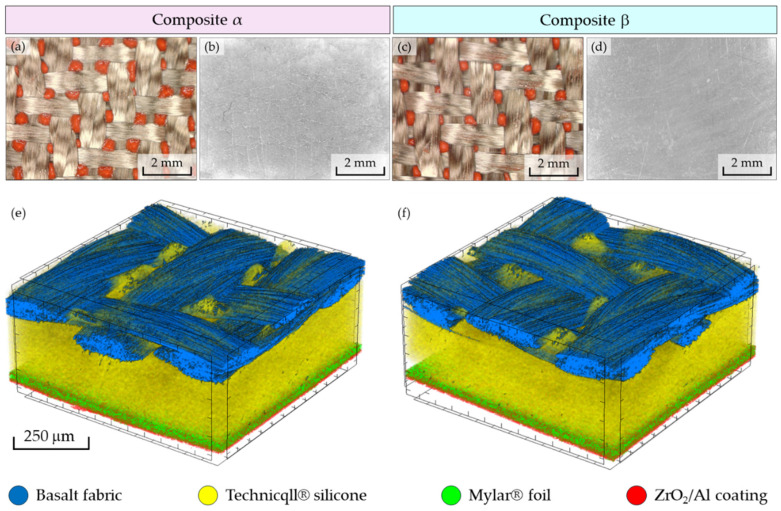
Optical microscopy images of the inner surface and outer surface of composite α (**a**,**b**) and composite β (**c**,**d**), micro-CT 3D visualization of composite α (**e**) and composite β (**f**)—composites area: 3 mm × 3 mm.

To characterize the structure of both composites, optical microscopy, OM (Delta Optical Smart 5MP PRO made by Delta Optical, Warsaw, Poland), which was used to take photos of the inner and outer layers of the composites (Figure 3a–d), and high-resolution micro-computed tomography, micro-CT (SkyScan 1272; Bruker, Kontich, Belgium), were used. The composites were scanned under the following conditions: X-ray source voltage: 50 kV, X-ray source current: 200 µA, pixel size: 4 µm, rotation: 180° rotation, rotation step: 0.2°, without filter. Using the software NRecon 1.7.4.2, CTAn 1.17.7.2+, and Data Viewer 1.5.6.2 software (made by Bruker, Billerica, MA, USA), the following parameters of composite layers were determined: (1) thickness, (2) total porosity, (3) yarn porosity (only for basalt fabric), and (4) thickness distribution of the ZrO_2_/Al coating on the fabric surface. Using the CTvox 3.3.0 r1403 software (made by Bruker), 3D visualizations of the composites with the all component layers were made (Figure 3e,f).

Thanks to the differences in X-ray absorption, all layers of the tested composites were identified. For better identification, they were highlighted with distinct colors in the 3D visualizations. However, due to the too small relative difference in X-ray absorption by ZrO_2_ and Al, it was not possible to distinguish the components of the ZrO_2_/Al coating (there was too little contrast between the signals coming from both coatings and those reaching the X-ray detector). An additional factor that made it difficult to distinguish the component layers of the ceramic–metal coating was their thickness and the fact that both coatings were supposed to be adjacent to each other. In the OM photos and micro-CT visualizations, it can be observed that in both composites, some of the silicone (marked in orange in the photos and yellow in the micro-CT visualizations) as a result of the applied mechanical load, penetrated to the other side of the fabric through the free spaces between the weft and warp yarns in the process of connecting with the basalt fabric (marked in brown in the OM photos and blue in the micro-CT visualizations). Based on the micro-CT visualization, it can also be observed that the layers composed of Technicqll^®^ silicone, Mylar^®^ foil, and the ZrO_2_/Al coating are free from cracks or cavities resulting from the formation of the composite, which can significantly affect the thermal insulation properties of the composites.

**Figure 4:**
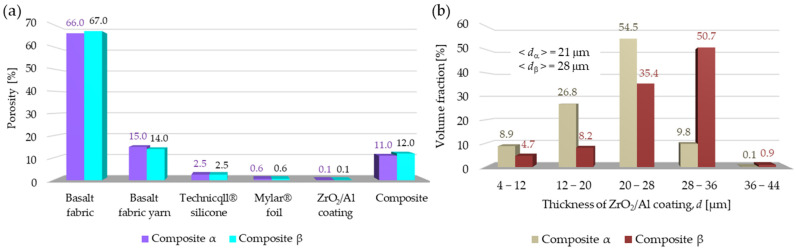
(**a**) Porosity of the tested composites; (**b**) distribution of the ZrO_2_/Al coating thickness in both composites.

Figure 4a shows the results of the porosity analysis of all layers that make up both composites. In the case of the basalt fabric, yarn porosity was additionally calculated. It should be noted that both calculated porosities refer to the fabric that is not yet a composite layer. As a result of forming the composite, some silicone penetrated the fabric, because of which both the total fabric porosity and the yarn porosity were partially reduced.

The results show that the porosities of the respective layers in both composites do not differ significantly (the difference in porosity between the respective components does not exceed 1%). Slight differences in porosity between the respective layers of the composites also translate into a small difference in the porosity of the entire composites, where the difference is 1%. These results indicate high uniformity of the layers that make up both composites as well as the repeatability of the composite manufacturing technology.

Figure 4b shows the distribution of the thickness *d* of the ZrO_2_/Al coating obtained for the PVD magnetron sputtering in which it was intended to achieve a 20 μm layer and a 30 μm layer on Mylar^®^ foil in composite α and composite β, respectively. Based on the distribution obtained for composite α, it can be observed that the largest volume part of the ZrO_2_/Al coating (54.5%) has a thickness ranging from 20 μm to 28 μm, while in the case of the composite β, the largest volume part of the ZrO_2_/Al coating (50.7%) has a thickness ranging from 28 μm to 36 μm. The average ZrO_2_/Al coating thicknesses determined based on the distributions are 21 μm for composite α and 28 μm for composite β. The calculated coating thicknesses do not differ significantly from the assumed coating thicknesses in both composites (20 μm for composite α and 30 μm for composite β). Small differences between the coating thickness assumed in the experiment and those calculated based on the micro-CT analysis indicate the correct calibration and optimization of the deposition process using PVD magnetron sputtering. On the other hand, the obtained wide coating thickness distributions in both composites may indicate uneven coating of the substrate with ZrO_2_/Al during deposition. When interpreting the coating thickness distributions, it should be borne in mind that their wide shape may be related to the substrate on which the coatings were deposited. Mylar^®^ foil is not a perfectly flat substrate with smoothness comparable to polished glass or a silicon wafer. It is rougher and has micro-cracks on its surface. As a result, the deposited material may settle on it less evenly. Therefore, there is a high probability that the use of alternative substrates with smaller surface defects will enable the deposition of a more even layer of ceramic–metal coatings. When choosing such a substrate, its appropriate resistance to high temperature, thickness and mechanical flexibility (ensuring appropriate ergonomics of work in a glove), and the possibility of its permanent connection with the adjacent internal layer of the composite (silicone) should also be taken into account. An alternative substrate may be the Kapton HN foil, which does not change its properties over a wide temperature range (−269 °C to 400 °C) and can be successfully laminated, metalized, or coated with adhesives [24,25].

#### 2.2.3. Evaluation of the Thermal Properties of Composites

**Figure 5:**
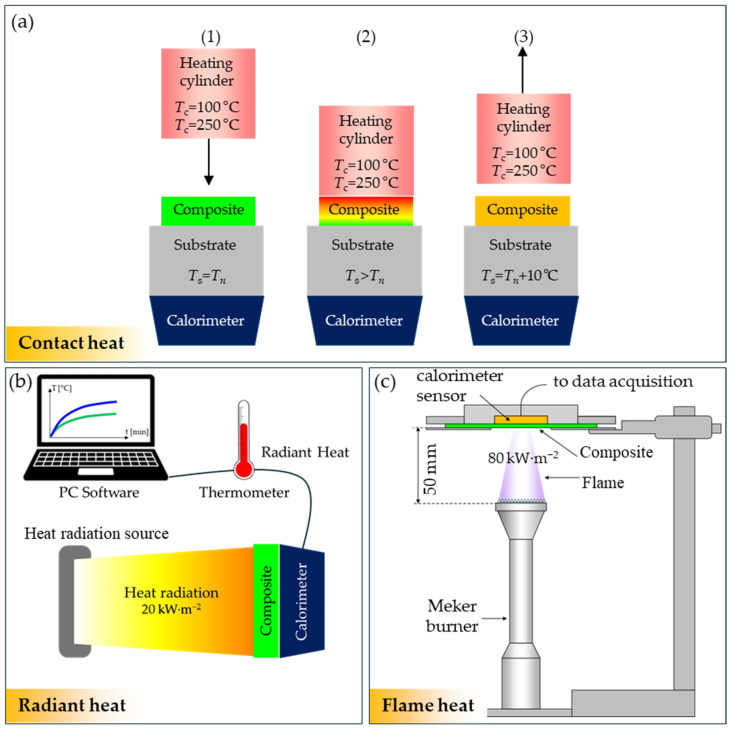
Schemes of three methods used to determine the influence of ZrO_2_/Al coatings on the resistance of composites to contact heat (**a**), radiant heat (**b**), and flame heat (**c**). The contact heat method consists of three stages with different substrate temperatures *T*_s_: (**a1**) *T*_s_ = *T*_n_; (**a2**) *T*_s_ > *T*_n_; (**a3**) *T*_s_ = *T*_n_ + 10 °C.

In order to investigate the thermal insulation properties of the composites under working conditions in thermal protective gloves, experiments were carried out in which they were exposed to *contact heat* (according to EN ISO 12127-1:2016-02 [26]), *radiant heat* (according to EN ISO 6942 [27]), and *flame heat* (according to EN ISO 9151 [28]). The schemes of the methods used are presented in Figure 5.

##### Contact Heat

The tests of the composites’ protection test against contact heat were carried out using an OTI thermal insulation tester (OTI Greentech AG, Berlin, Germany) for contact temperatures of 100 °C and 250 °C. Figure 5 shows a diagram of the composite protection test against contact heat performed for the purposes of the tests described in the article. The composite placed on the substrate is contacted with the ZrO_2_/Al coating with a cylinder heated to the contact temperature *T*_c_ (100 °C or 250 °C). In the first stage of the test, the substrate temperature *T*_s_ is equal to the ambient temperature *T*_n_ (Figure 5(a1)). As a result of heat flow from the cylinder through the composite, the substrate temperature *T*_s_ increases (Figure 5(a2)). In the experiment, the threshold time *t*_t_ is calculated, which is measured from the first contact with the cylinder until the moment when the calorimeter temperature increases by 10 °C from the initial value *T*_s_ = *T*_n_ + 10 °C (Figure 5(a3)). Based on the performed experiment, protective gloves are classified into one of four performance levels of contact heat protection. Subsequent performance levels are achieved when the threshold time *t*_t_ ≥ 15 s is reached at four increasingly higher contact temperatures *T*_c_ = 100 °C, 250 °C, 300 °C, and 500 °C (Table 2).

##### Radiant Heat

Figure 5b shows a scheme of the method for evaluating the protection of composites against radiant heat. During the experiment, the composite is placed on the calorimeter in such a way that the basalt fabric is directly adjacent to the calorimeter, while the ZrO_2_/Al coating is exposed to the source of thermal radiation with a heat flux density of 20 kW m^−2^. As a result of the incidence of thermal radiation on the ZrO_2_/Al coating, the remaining composite layers, up to the basalt fabric, heat up. During the test, the Radiant Heat Transfer Index (*RHTI*_24_) (time to achieve a temperature rise of 24 °C in a calorimeter when evaluating the sample with a specified incident heat flux density—this temperature rise indicates that the gloves user experienced second-degree burns) was determined. Depending on the *RHTI*_24_ value, the gloves are classified into one of four performance levels of protection (Table 2).

##### Flame Heat

Figure 5c illustrates the experimental setup used to measure the protection of composites against flame heat. It consists of a Meker gas burner, a copper disk calorimeter, a thermometer, and dedicated software. During the experiment, a horizontally oriented test sample is subjected to an incident heat flux from a gas burner flame placed underneath it. The heat is incident on the outer surface of the sample (ZrO_2_/Al coating) and passes through the subsequent composite layers to the basalt fabric to which the calorimeter is attached. The calorimeter was connected to a digital, computer-controlled thermometer that recorded the temperature increase in the basalt fabric over time. The tests were carried out for a density of the incident heat flux of 80 kW m^−2^. Based on the conducted experiment, Heat Transfer Index *HTI*_24_ (time to achieve a temperature rise of 24 °C in a calorimeter when evaluating the sample with a specified incident heat flux density—this temperature rise indicates that the gloves user experienced second-degree burns) was determined, which means the average time needed to achieve a temperature increase of 24 °C in the calorimeter. Depending on the *HTI*_24_ value, the gloves are classified into one of four performance levels of protection (Table 2).

## 3. Results and Discussion

**Figure 6:**
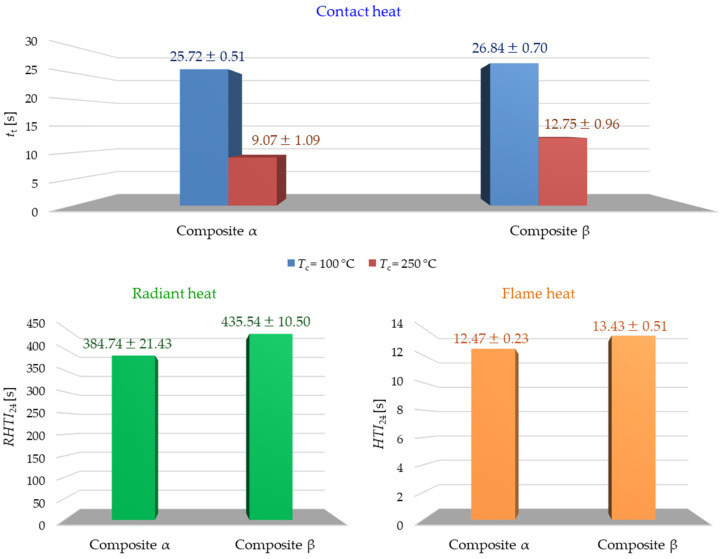
Results of the three methods used to determine the influence of ZrO_2_/Al coatings on the resistance of composites to contact heat, radiant heat, and flame heat.

Figure 6 presents the results of tests on the thermal insulation properties of composites exposed to contact heat, radiant heat, and flame heat.

According to the results of the experiment using *contact heat* for both contact temperatures, *T*_c_ (100 °C and 250 °C), the threshold time of the composite β is longer than the *t*_t_ of composite α (by 1.12 s and 3.68 s, respectively). According to EN ISO 12127-1:2016-02 [26], both composites can be used for thermal protective gloves only up to a contact temperature of 100 °C (first performance level), for which the threshold time *t*_t_ exceeds 15 s.

The results of the experiment using *radiant heat* showed that composite β is characterized by a higher *RHTI*_24_ value than the composite α by 50.80 s. According to PN-EN 407:2020-10 [29], both composites can be used for thermal protective gloves, and they reach the fourth performance level for which the *RHTI*_24_ exceeds 95 s.

The results of the tests using *flame heat* showed that the *HTI*_24_ value for composite β is 0.96 s higher compared to composite α. According to PN-EN 407:2020-10 [29], both composites can be used for thermal protective gloves, and they reach the third level of effectiveness for which the *RHTI*_24_ exceeds 10 s. Taking into account the classification of performance levels for the tested composites obtained on the basis of the obtained test results, it can be stated that the composites can be used in gloves dedicated to steelworkers, welders, and metallurgists; however, only in the back part, due to the results of tests on resistance to contact heat, they cannot be used in the palm part of the glove protecting against temperatures higher than 100 °C.

Comparing the obtained results of resistance to contact heat and thermal radiation to the literature results [4,7], the composites produced for use in protective gloves obtained the same fourth (highest) level of protection against thermal radiation. In the case of resistance to contact heat, similarly to the literature [4,7], the first performance level was obtained (for *T*_c_ = 100 °C) for both composites. However, the β composite for *T*_c_ = 250 °C reached *t*_t_ = 12.75 s, which is a promising result, and perhaps another modification using the PVD technique will allow obtaining resistance to a contact temperature of 250 °C. It should be remembered that we compared the results of composites with the produced variants of protective gloves. According to the literature, these gloves were produced in a multilayer structure with other components.

In the graphs of Figure 6, next to the measured values, the standard deviations obtained in *t*_t_, *RHTI*_24_, and *HTI*_24_ measurements are also given. Depending on the method used, the deviation of specific measurement results from the average value is different. In the case of the *contact heat* method for *T*_c_ = 100 °C, the standard deviation does not exceed 3% of the average value *t_t_*, while for *T*_c_ = 250 °C, it does not exceed 12% of the average value *t*_t_. Moreover, for *T*_c_ = 100 °C, both composites obtain very similar *t_t_* values (the difference is 4% in favor of the composite β and is close to the standard deviation value), while for *T*_c_ = 250 °C, the difference in *t_t_* is more pronounced and is 29% in favor of the composite β. For the *radiant heat* method, the standard deviation does not exceed 6% of the *RHTI*_24_ mean value, while for the flame heat method, the standard deviation does not exceed 4% of the *HTI*_24_ mean value. The differences described in measurement uncertainty may be caused by many factors related to both the method used as described in the standard (number of measurements, accuracy of the measuring device) and the tested samples (uniformity of the composite structure).

Summarizing the results of all three methods used, it can be seen that the β composite (with a thicker ZrO_2_/Al coating of 30 µm) is characterized by better thermal insulation properties than the α composite (with a thinner ZrO_2_/Al coating of 20 µm). It can be assumed that this is due to the twice as thick ZrO_2_ coating in the β composite with thermal insulation properties. However, it should be remembered that both composites differ slightly in terms of morphology (thickness of all four layers, their uniform structure throughout the composite, their interconnection, and porosity). All these parameters can also affect the results of the three experiments.

Although the authors in this article focused exclusively on the functional tests of the thermal insulation properties of the tested composites, it should be realized that in the full assessment of whether the tested composites provide effective protection for the user against harmful factors of a hot environment, their durability during use should be taken into account. Protective gloves used in high ambient temperatures are also exposed to a wide range of mechanical impacts related to the specifics of the user’s work. This is, for example, the cyclic bending process when gripping various objects such as tools, machine elements, and construction elements. If these objects are also sharp, the glove is, in addition to friction, also exposed to puncture or cutting. Cyclic mechanical impacts can lead to damage to the composite due to the fatigue of the materials from which it is made. For example, the glove may significantly lose its protective properties against radiant heat due to damage resulting from abrasion of the ZrO_2_/Al coating reflecting infrared waves. In this case, it is necessary to take care of both the mechanical resistance of the ceramic–metal coating itself and its adhesion to the substrate. The ability to reflect infrared radiation from the ZrO_2_/Al coating is determined not only by its thickness but also by its roughness. The higher the roughness, the lower the ability to reflect thermal radiation from the outer surface of the composite due to the lower emissivity value. Roughness reduction can be achieved by using a smooth substrate, which, in the case of the tested composites, was Mylar^®^ foil. In another example case, as a result of deep puncture with a sharp tool, the thickest layer of the composite (made of silicone) can be damaged, which will contribute to a significant deterioration in the performance level of protection of the glove for contact heat. An important factor determining the mechanical strength of the gloves is the suitably durable connection of the individual composite layers so that they do not delaminate or move relative to each other during use.

## 4. Conclusions

The research presented in this article describes the evaluation of the use of two four-layer textile composites, α and β, with ZrO_2_/Al coatings of different thicknesses (deposited by PVD magnetron sputtering) with potential use in thermally insulating protective gloves used in metallurgy or steelmaking. The structure of the composites was analyzed using high-resolution X-ray microtomography, while their assessment in terms of the safety of the glove user was conducted based on experiments in which they were exposed to contact heat, radiant heat, and flame heat. The following conclusions can be drawn from the obtained results:

- The analysis of the structure of both composites using micro-CT showed that in both tested composites the arrangement and morphology of the component layers is comparable, which proves the homogeneity of the raw materials used and the repeatability of composite formation.
- The performed micro-CT structural analysis of the ZrO_2_/Al coatings showed that their thicknesses determined in the PVD magnetron sputtering process did not differ significantly from the actual coating thicknesses obtained in both composites. The difference in the assumed and actual average thickness of the ceramic–metal coating was 5% for composite α and 7% for composite β.
- The results of the experiment in which the composites were exposed to *contact heat* show that both composites can be used for thermal protective gloves only up to a contact temperature of 100 °C (first performance level).
- The results of the experiment in which the composites were exposed to *radiant heat* show that both composites can be used for thermal protective gloves, and they reach the fourth performance level.
- The results of the experiment in which the composites were exposed to *flame heat* show that both composites can be used for thermal protective gloves, and they reach the third performance level.
- Summarizing the results of the three experiments, it can be stated that the presented four-layer textile composites equipped with ZrO_2_/Al coatings provide effective protection against contact heat, radiant heat, and flame heat and can be successfully used in the construction of the back part of metallurgical protective gloves.

## Figures and Tables

**Table 1:** Characteristics of the two tested composites.

Composite	Layer	Composition	Layer/Composite Thickness [μm]	
α	Fabric	Basalt	380	1315
	Silicone	Technicqll^®^	830	
	Foil	Mylar^®^	85	
	PVD Coating	ZrO_2_/Al	10/10	
β	Fabric	Basalt	380	1325
	Silicone	Technicqll^®^	830	
	Foil	Mylar^®^	85	
	PVD Coating	ZrO_2_/Al	20/10	

**Table 2:** Performance levels for contact heat protection (according to EN ISO 12127-1:2016-02 [26]), radiant heat protection (according to PN-EN 407:2020-10 [29]), and flame heat protection (according to PN-EN 407:2020-10 [29]).

Performance Level	Contact Heat		Radiant Heat	Flame Heat
	*T*_c_ [°C]	*t*_t_ [s]	*RHTI*_24_ [s]	*HTI*_24_ [s]
1	100	≥15	≥7	≥4
2	250	≥15	≥20	≥7
3	350	≥15	≥50	≥10
4	500	≥15	≥95	≥18

## Data Availability

The original contributions presented in this study are included in the article. Further inquiries can be directed to the corresponding authors.
